# Edge Computing and Fault Diagnosis of Rotating Machinery Based on MobileNet in Wireless Sensor Networks for Mechanical Vibration

**DOI:** 10.3390/s24165156

**Published:** 2024-08-09

**Authors:** Yi Huang, Shuang Liang, Tingqiong Cui, Xiaojing Mu, Tianhong Luo, Shengxue Wang, Guangyong Wu

**Affiliations:** 1 School of Intelligent Manufacturing Engineering, Chongqing University of Arts and Sciences, Chongqing 402160, Chinacuitq@cqu.edu.cn (T.C.); 20230014@cqwu.edu.cn (S.W.); 20070110@cqwu.edu.cn (G.W.); 2 Key Laboratory of Optoelectronic Technology & Systems, Ministry of Education, International R & D Center of Micro-Nano Systems and New Materials Technology, Chongqing University, Chongqing 400044, China; 3 Department of Information and Intelligence Engineering, Chongqing City Vocational College, Chongqing 402160, China; 4 Mining Industry Digital Transformation Laboratory, Mining Institute, T.F. Gorbachev Kuzbass State Technical University, 28 Vesennya St., 650000 Kemerovo, Russia

**Keywords:** mechanical equipment monitoring, wireless sensor networks, mobileNet, fault diagnosis, edge computing

## Abstract

With the rapid development of the Industrial Internet of Things in rotating machinery, the amount of data sampled by mechanical vibration wireless sensor networks (MvWSNs) has increased significantly, straining bandwidth capacity. Concurrently, the safety requirements for rotating machinery have escalated, necessitating enhanced real-time data processing capabilities. Conventional methods, reliant on experiential approaches, have proven inefficient in meeting these evolving challenges. To this end, a fault detection method for rotating machinery based on mobileNet in MvWSNs is proposed to address these intractable issues. The small and light deep learning model is helpful to realize nearly real-time sensing and fault detection, lightening the communication pressure of MvWSNs. The well-trained deep learning is implanted on the MvWSNs sensor node, an edge computing platform developed via embedded STM32 microcontrollers (STMicroelectronics International NV, Geneva, Switzerland). Data acquisition, data processing, and data classification are all executed on the computing- and energy-constrained sensor node. The experimental results demonstrate that the proposed fault detection method can achieve about 0.99 for the DDS dataset and an accuracy of 0.98 in the MvWSNs sensor node. Furthermore, the final transmission data size is only 0.1% compared to the original data size. It is also a time-saving method that can be accomplished within 135 ms while the raw data will take about 1000 ms to transmit to the monitoring center when there are four sensor nodes in the network. Thus, the proposed edge computing method shows good application prospects in fault detection and control of rotating machinery with high time sensitivity.

## 1. Introduction

Advanced equipment serving as the backbone of the national economy and national defense has become increasingly complex and intelligent. Among these, rotating machinery—an essential and pivotal component of modern industrial machinery—operates in environments characterized by extreme conditions such as high temperature, high pressure, high speed, and enduring alternating loads [1,2,3]. Given the prolonged exposure to such an operating environment, these machines are susceptible to failures that not only result in substantial economic losses but also pose a risk of severe safety incidents. Moreover, the complicated architecture of advanced mechanical equipment leads to the difficulty of the decision-making process for maintenance [4]. The assurance of security, reliability, and informed maintenance decisions for rotating machinery remains a formidable challenge in the field of prognostics and health management [5,6,7]. To address this challenge, the integration of edge computing and intelligent fault diagnosis emerges as a promising methodology. This approach holds the potential to guarantee the intelligent operation and maintenance of advanced equipment, thereby mitigating risks and enhancing overall system performance [8].

In contrast to conventional wired acquisition systems, mechanical vibration wireless sensor networks (MvWSNs) are a self-organizing distributed system coupled with the capacity for on-chip processing, which is a near-sensing monitoring system [9,10,11]. This unique characteristic allows them to be seamlessly deployed in rotating and sealed environments for efficient data collection without posing risks to the monitoring equipment, such as those found in wind power gearboxes.

Typically comprising processors with computing capabilities like STM32 [12] or MSP430 [13], MvWSNs operate with limited processing power and is considered an edge platform in comparison to high-performance computers. In addition, MvWSNs collect a large amount of vibrational data in a short period of time. The sheer volume of data generated in these short intervals not only highlights the processing constraints but also amplifies the communication burden placed on the network [14]. For instance, at a sampling frequency of 25,600 Hz and utilizing a 24-bit analog-to-digital converter (ADC), MvWSNs can generate a substantial 75 kB of data in just one second. The limited bandwidth of existing MvWSNs lead to data blocking once the sampling data is simultaneously uploading due to the limited bandwidth of the MvWSNs, resulting in a large amount of energy consumption. In previous research [15], data compression for MvWSNs is proposed to address the issue of mass data, but the uploaded data is still up to 50% of the original data.

The advancement of deep learning (DL) has ushered in the development of lightweight models, offering a promising avenue for integrating edge computing with DL in MvWSNs. This integration aims to deploy the fault detection model on MvWSNs nodes, reducing the size of data transmissions and facilitating prompt decision making. Such an approach holds the potential to elevate the real-time performance of data processing within MvWSNs [16].

Existing intelligent fault diagnosis models primarily target high-performance computers, and are incompatible with the computing and storage constraints of the MvWSNs. This incompatibility stems from several key challenges: (1) Excessive model parameters: existing fault diagnosis models, designed for high-performance processors, prioritize detection accuracy during training, resulting in the generation of a substantial number of model parameters. (2) Limited on-chip resources: the MvWSN nodes possess constrained on-chip resources, making it challenging to load and calculate an extensive set of model parameters simultaneously. (3) Floating-point calculation constraints: the micro center unit (MCU) in MvWSNs often grapple with limited floating-point calculation capabilities, especially when dealing with complex computations, such as multiplication and division. Model parameters, typically represented as double precision floating-point numbers, exacerbate these challenges.

To address the problem of DL models with many parameters, long operation times and, difficulties in running in the MvWSNs, an improved and lightweight MobileNet fault diagnosis method was proposed. The main contributions are as follows:

(1) The operational status of rotating machinery is acquired noninvasively, eliminating the need for intricate wired cable installations and averting any damage to the monitoring equipment.
(2) Inspired by the small and lightweight DL model for mechanical equipment monitoring, a lightweight compressed MobileNet for MvWSNs senor node is proposed.
(3) The proposed method was tested on a sensor node to demonstrate its efficiency on a source-constrained embedded platform, including model size, time efficiency, and accuracy.

The rest of the paper is organized as follows: Related work and motivation of lightweight DL model are analyzed in Section 2. Section 3 introduces the design of the optimized MobileNet model and its deployment in MvWSNs. The experiments are set to analyze the performance in Section 4. Finally, Section 5 concludes this paper.

## 2. Related Work

Nowadays, researchers pay attention to the lightweight model for near-sensing applications and they make efforts to design CNN-based modes to improve the performance of the model while reduing the parameters and the computing costs of the model. In this way, the model can be implemented on source-constrained edge computing platforms. So, in this section, we will introduce the related work on the development of a mobileNet model in order to provide a deeper understanding of the lightweight model.

### 2.1. Lightweight Deep Learning Model

The classical machine learning (ML) methods have been extensively adopted in machine feature extraction and fault information recognition before the widespread adoption of DL methods. In contrast to DL models that often have massive parameters, classical ML models demand lower computational costs. This characteristic makes them well-suited for deployment on edge nodes, which typically have limited computing resources [17]. Even though the classical ML methods for fault diagnoses can achieve good application results and occupy less memory space, there are still two major issues: (1) Traditional signal processing techniques often rely heavily on the prior knowledge and expertise of domain specialists. The selection and design of feature extraction methods are guided by the insights and understanding of these experts. (2) The fault classifiers built upon extracted features may not be universally applicable across different applications or scenarios.

Recently, there has been a swift and robust development of DL methods in the field of machine fault diagnosis applications. On the other hand, edge computing, as a novel computing paradigm, has been introduced recently to specifically tackle the challenge posed by substantial data transmissions and their potential impact on energy consumption in MvWSNs. Therefore, a combination of edge computing and DL shows great potentials in real-time machine signal processing and fault recognition, which is helpful to reduce the size of the transmission data and allow the control of rotation machinery with high time sensitivity [18].

To execute DL models on edge devices with limited computational power and memory, it is essential to prune and optimize the models to minimize the number of parameters. Park et al. [19] proposed a lightweight real-time fault detection method named LiReD and the model was deployed on a Raspberry Pi for an industrial robot manipulator, which enables the monitoring system to perform necessary detection and control tasks in a short time. Lu et al. [20] proposed an in situ motor fault diagnosis method via implementing an enhanced CNN on a Raspberry Pi sensor node, an edge platform with up to 8-GB memory and a 1.5-GHz Quad-Core 64-bit CPU. The data acquisition and data processing were executed on the edge node for in situ fault diagnosis. Malviya et al. [21] proposed a lightweight convolutional autoencoder implemented on a low-cost FPGA platform to discern anomalies in vibration data sets. According to above analysis, numerous DL methods for edge device are proposed; however, these edge devices are still operating on the high-performance platform.

The nodes in MvWSNs are expected to process the essential capabilities for acquisition tasks while minimizing energy consumption for long-term monitoring. Therefore, the existing MvWSNs node is occupied with a STM32 MCU, which has only less than 1 MB of memory and the core frequency is usually less than 200 MHz [22]. With the demands of DL models for source-constrained edge devices, some lightweight networks have been emerged, such as MobileNet [23], SqueezeNet [24], and ShuffleNet [25], and been proposed successively, wherein MobileNet is a typical lightweight CNN model. The research found that MobileNet V1 can significantly reduce the model size while ensuring accuracy.

Yu et al. [26] proposed an end-to-end intelligent diagnosis method for bearings based on MobileNet V1. Pham et al. [27] established a lightweight model for bearing fault diagnosis based on MobileNet V2 to optimize the requirements of the system resources. Yao et al. [28] used a butterfly-transform module to replace the pointwise convolution based on MobileNet V3, which can achieve high efficiency and high accuracy. Among them, MobileNet V1 is the most promising model for MvWSNs node due to its simple processing.

### 2.2. MobileNet

The MobileNet network [23] is a lightweight architecture that embraces depthwise separable convolutions (DSC) instead of conventional convolutions. This innovative approach not only maintains model accuracy but also markedly diminishes the number of parameters and computational requirements. As a result, the reliance on hardware computing resources is substantially reduced. MobileNet is currently recognized as an efficient and lightweight architecture, particularly well-suited for embedded devices with limited computing resources.

**Figure 1:**
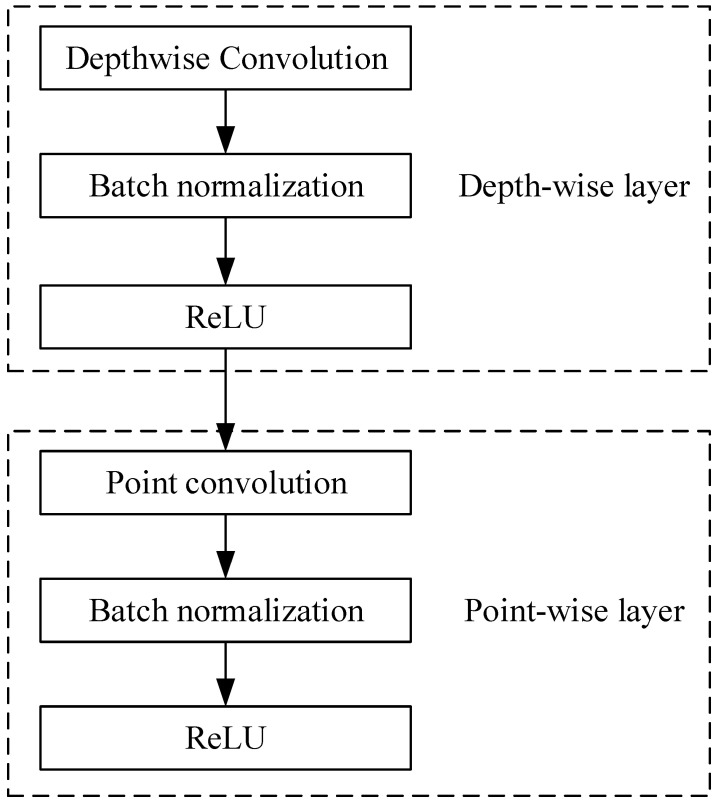
The basic convolutional structure of MobileNet.

**Figure 2:**
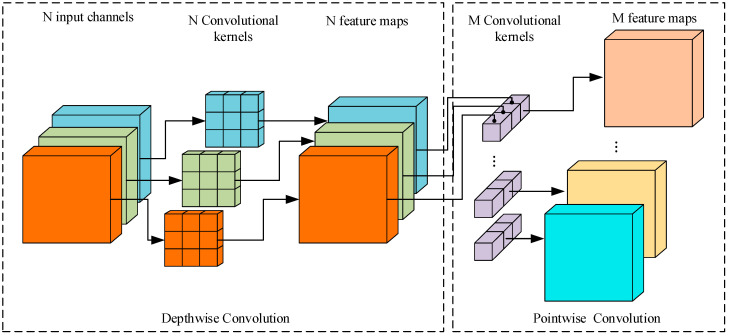
The depthwise separable convolution structure.

As shown in Figure 1 and Figure 2, the depth separation convolution is divided into depthwise convolution (DW) and pointwise convolution. Depthwise convolution only has a one-dimensional convolution kernel, which is not extended after completing the convolution. To be specified, let us assume that the input channels and output channels are represented by *M* and *N*, respectively. The kernel size is $D_{k}\times D_{k}$ and the input map size is $D_{f}\times D_{f}$. The cost of $C_{dw}$ is considered as follows:

(1) $$C_{dw}=D_{k}\times D_{k}\times M\times D_{f}\times D_{f}$$

And the cost of the standard convolution is expressed as follows:

(2) $$C_{std}=D_{k}\times D_{k}\times M\times N\times D_{f}\times D_{f}$$

Pointwise convolution uses a 1×1 convolution kernel to convolute only one region, combining the features of each channel to achieve less computational and model parameter requirements. The cost of a point convolution can be denoted as

(3) $$M\times N\times D_{f}\times D_{f}$$

Consequently, the overall cost $C_{dws}$ is represented as follows:

(4) $$C_{dws}=D_{k}\times D_{k}\times M\times D_{f}\times D_{f}+M\times N\times D_{f}\times D_{f}$$

Then the ratio between $C_{std}$ and $C_{dws}$ is as follows:

(5) $$\begin{array}{lll} \frac{C_{dws}}{C_{std}} & = & \frac{D_{k}\times D_{k}\times M\times D_{f}\times D_{f}+M\times N\times D_{f}\times D_{f}}{D_{k}\times D_{k}\times M\times N\times D_{f}\times D_{f}}\\ & = & \frac{1}{N}+\frac{1}{D_{k}^{2}} \end{array}$$

Equation (5) indicates that if the convolution kernel is set to 3×3, the cost of DSC can be reduced by about nine times compared to the standard convolution, which proves that the separation of convolutions is helpful to lower the computational cost and that MobileNet is a lightweight architecture. Such a lightweight architecture is hopefully applied on source-constrained MvWSNs.

## 3. Proposed Method

### 3.1. Proposed Lightweight Model

**Figure 3:**
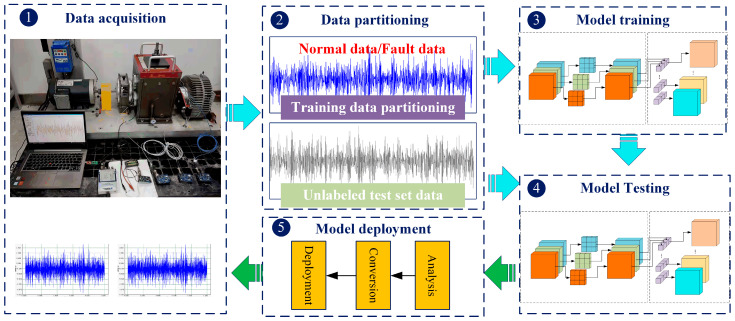
Flowchart of the proposed method for MvWSNs.

Faced with the requirements of deploying the DL model in MvWSNs, a lightweight network is proposed based on MobileNet V1. Figure 3 describes the steps of the proposed method for MvWSNs. The specific steps are follows:

Step 1: Vibration signal acquisition. The MvWSNs node is deployed in the drivetrain diagnostic simulator (DDS) to collect the vibration signals of the X and Y axes of the input and output axes.
Step 2: Model training and dataset partitioning. The collected vibration data is divided into multiple samples with a sample length of 1024. Then, the samples are divided into training set data and testing set data. The training set data is annotated with five samples: normal (N), gear pitting (F1), root crack (F2), bearing outer ring fault (F3), and inner ring fault (F4). Note that the test set data is not labeled with samples.
Step 3: Model construction and offline training. The limited computing resources of the MvWSNs node is unable to support model training. Therefore, the model training is still being conducted on a high-performance computer.
Step 4: Online testing of model accuracy. Unknown test set data is inputted into the lightweight model trained in the previous step for fault diagnosis and classification, and the accuracy of diagnostic testing is calculated.
Step 5: Model deployment. The model is deployed to MvWSNs nodes for online monitoring and fault diagnosis.

The proposed network structure of the lightweight fault diagnosis model based on MobileNet is shown in Table 1.

### 3.2. Model Deployment in MvWSNs

The MvWSNs node is composed of two main parts. A transmission chip (CC2530) is mainly responsible for network communication and management. The main microprocessor STM32F405 (STMicroelectronics International NV, Geneva, Switzerland) is adopted for data acquisition and data processing. CC2530 integrates an enhanced 8051 but it can only deal with some basic computation. The core of STM32F405 is a 32-bit Cortex-M4 CPU with FPU and its flash and SRAM memory can be up to 1 M byte and 196 k bytes, respectively. Even though the computing and storage resources are limited, it still can handle floating-point and matrix operations, which provides the foundation condition for the hardware for the lightweight model.

In addition, STMicroelectronics provide a tool named STM32Cube.AI (version 7.0.0), which enables the deployment of DL networks on the microcontrollers [29]. It supports a series of trained networks from several mainstream frameworks, including TensorFlow Lite, Keras, qKeras, or Pytorch. Also, a wide range of DL networks are supported, such as multi-layer perceptron and convolutional neural networks, including residual neural networks.

As for an imported model, it is necessary to first analyze the correctness of the model, and then analyze the input data size, output data size, required computational complexity, weight size, and activation function size of the network. Based on STM32Cube.AI, the proposed network is shown in detail. Finally, the required computing and model-occupied memory, including Flash and RAM, is analyzed to determine whether the model can be deployed on the MCU or not. For the proposed model, the analysis results are shown in Table 2.

## 4. Experiments and Analysis

### 4.1. Performance on Open Data

The performance of the proposed model was evaluated using open vibration data sets, which can be acquired from the Case Western Reserve University (CWRU) Bearing Data Center (accessed on 12 May 2023, https://engineering.case.edu/bearingdatacenter). Data were collected at various locations, including the drive end, fan end, and base, with defect depths of 0.007 inches, 0.014 inches, and 0.021 inches. The CWRU dataset comprises drive-end-bearing data at 12 kHz and 48 kHz and fan-end-bearing data at 12 kHz.

This study focused on the drive-end data with a sampling frequency of 12 kHz. Four data are adapted: normal (N), ball fault (BF), bearing outer ring fault (OF), and inner ring fault (IF). The vibration signals were preprocessed and divided into training and testing sets, with each sample containing 1024 points without overlap. Each class contains 100 samples, and the training data ratio was set at 60%.

**Figure 4:**
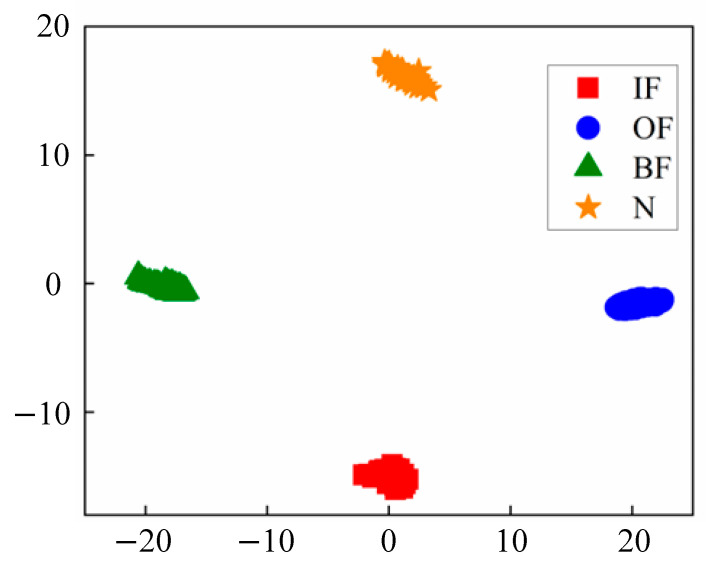
TSNE visualization results of CWRU data sets.

**Figure 5:**
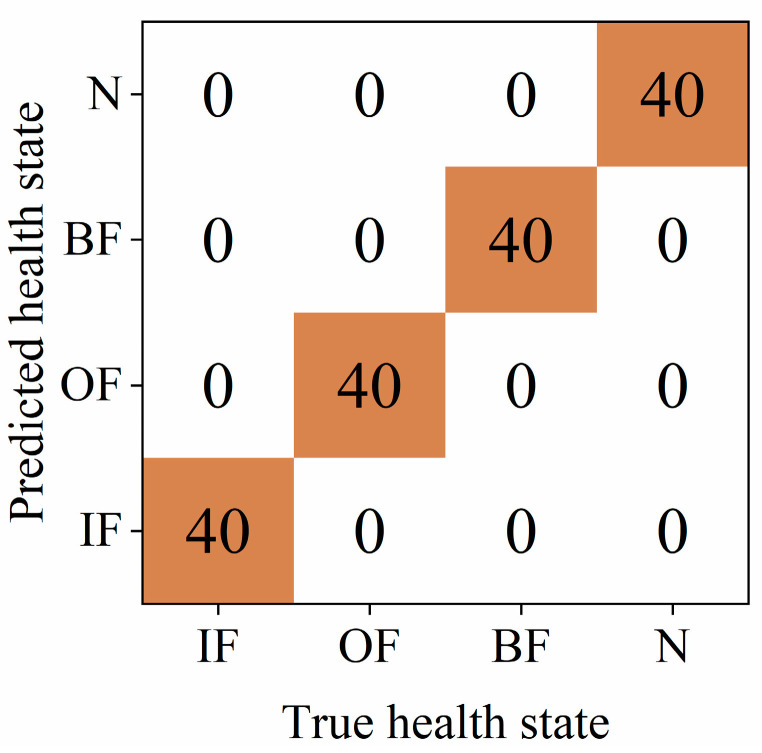
The confusion matrix results of CWRU data sets.

The experimental results are shown in Figure 4 and Figure 5. As can be seen from the results, the four-condition data are classified clearly. The confusion matrix results also demonstrate the good performance of the proposed model.

### 4.2. Fault Simulator Dataset of DDS

DDS is an experimental object, consisting of a two-stage planetary gearbox and a two-stage fixed shaft gearbox. The gears and transmissions of each gear on the experimental platform are shown in Table 3.

**Figure 6:**
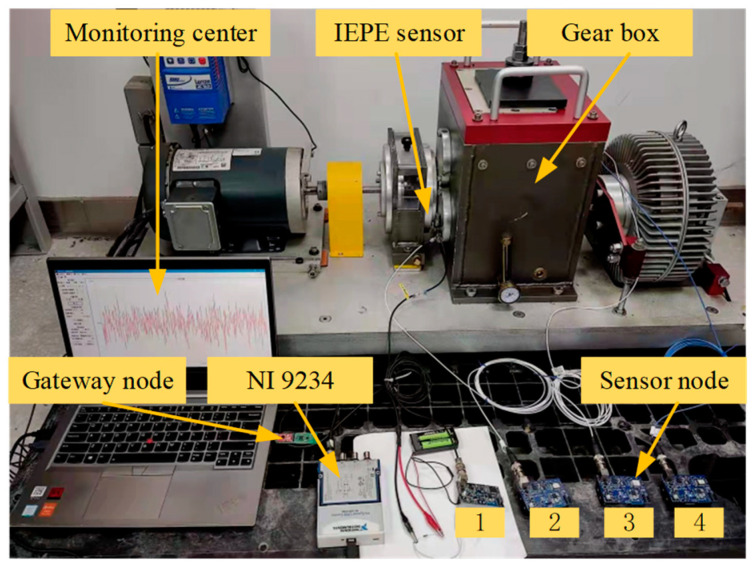
Conditions of DDS in the experiments.

The layout of the DDS test bench status monitoring is shown in Figure 6, which consists of four acquisition nodes (node numbers 1–4). Nodes 1 and 2 are connected to IEPE sensors to collect vibration signals in the X and Y directions of the intermediate shaft. Nodes 3 and 4 are connected to IEPE sensors to collect vibration signals in the X and Y directions of the output shaft.

### 4.3. Model Training

**Figure 7:**
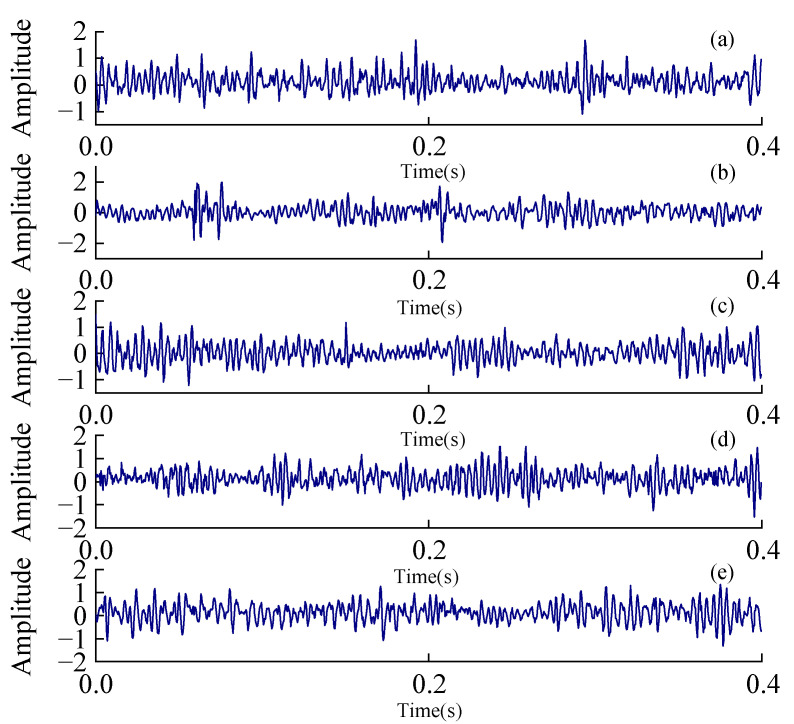
Vibration signals in different conditions from MvWSNs sensor node: (**a**) Normal; (**b**) Tooth surface pitting fault of gear; (**c**) Tooth root crack fault of gear; (**d**) Outer fault of bearing; (**e**) Inner fault of bearing.

In the experiments, five kinds of states of planetary gear boxes are designed, including two kinds of gear faults, two kinds of bearing faults, and a normal state. The description of each condition of planetary gear is shown in Table 4. The sampling frequency is set to 2560 Hz and the sampling length is 20,480. For each condition, we collect 40 sets of condition data. The original data is divided into 800 samples according to a sampling length of 1024, and the number of training sets and testing sets are 600 and 200, respectively. The raw time waveform of each health condition is shown in Figure 7.

During training, cross entropy is considered as the loss function. The initial learning rate is set to 0.001, and the weights and biases are using the stochastic gradient descent optimizer with a batch size of 50. The total training epochs are set to 50. After each convolution operation, batch normalization is performed, as shown in Figure 1, which is able to prevent gradient dispersion and improve the generalization ability of proposed model.

### 4.4. Testing Results

**Figure 8:**
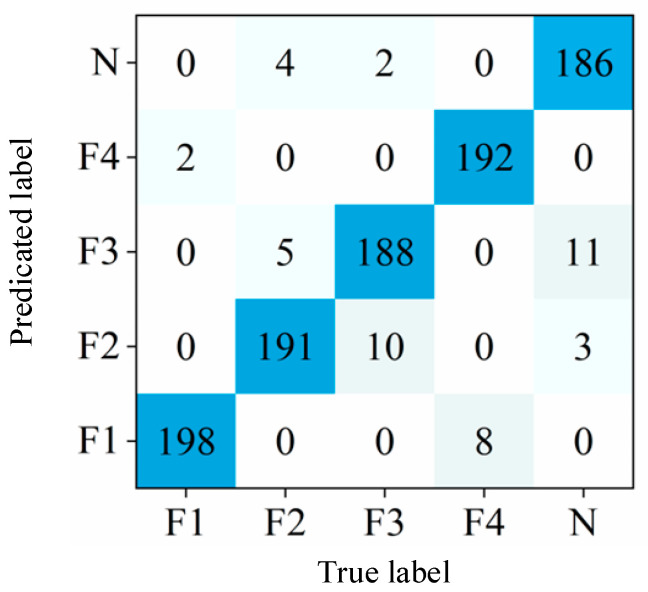
The confusion matrix results.

After training the model on a high-performance computer, the test sample of 1000 unknown labels are tested via the training model, then the testing results are compared with the real labels. Based on the test results, a confusion matrix was used to evaluate the classification accuracy and visualization of the test results, as shown in Figure 8. The prediction accuracy ranged from 93% to 99%, indicating the effectiveness of the proposed lightweight MobileNet network in classifying normal states and fault conditions.

**Figure 9:**
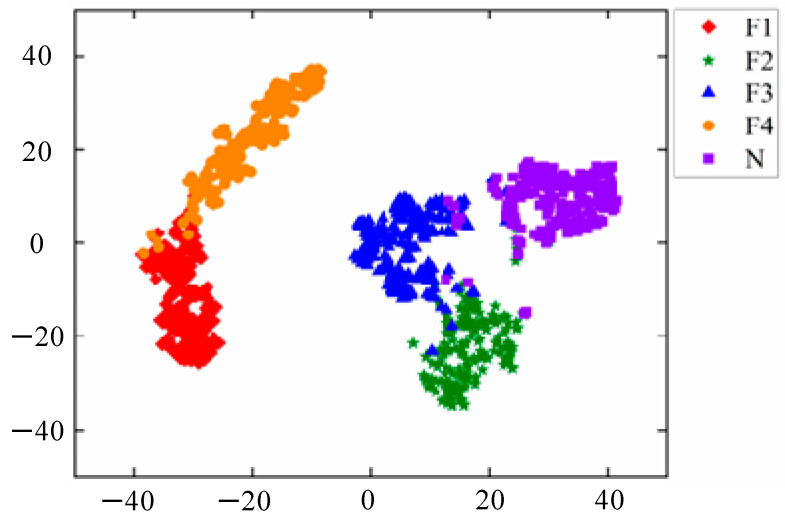
TSNE visualization results.

The t-distribution random neighborhood embedding t-SNE is used to reduce the dimensionality and visually evaluate the quality of the learned high-dimensional features. The proposed method’s feature clustering visualization results after the t-SNE dimensionality reduction are shown in Figure 9. From the figure, it can be seen that only a small number of samples are misclassified, and the model’s feature clustering effect is good, with only F3 faults overlapping more with N and F2 faults.

As a result of the experiments, the proposed lightweight model was evaluated by open vibration data sets and simulated data sets, these two data sets contain bearing fault data under various conditions. Both experimental results demonstrated the good classification performance of the proposed model, which indicates the good generalization performance of fault diagnosis models.

### 4.5. Comparison Result on Mechanical WSN

**Figure 10:**
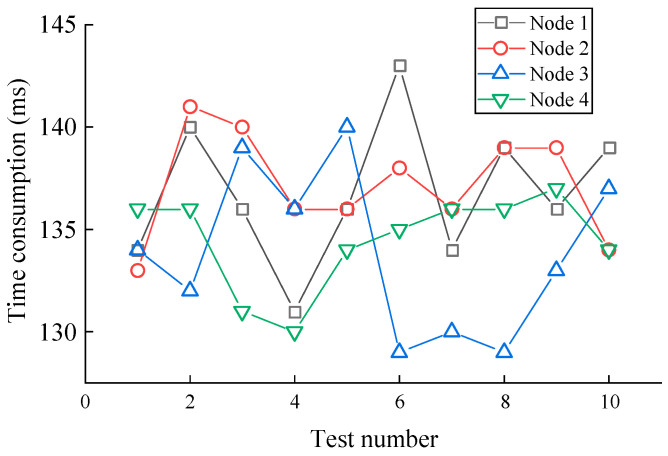
The results of time consumption of four nodes.

The modified MobileNet model was analyzed by STM32Cube.AI and deployed on the MvWSNs nodes. Firstly, the time consumption of the computation operation was evaluated. Figure 10 shows the time consumption results of the monitoring data over 10 tests. The average time consumption for obtaining the diagnostic results is about 136 ms.

The diagnostic accuracy for each condition via on-chip computing of a sensor node is shown in Table 5. The average accuracy is up to 98%. Compared to the transmitted raw vibration data, there is only about 5 Bytes to transmit to the monitoring center, which is about 0.1% of the original data.

**Figure 11:**
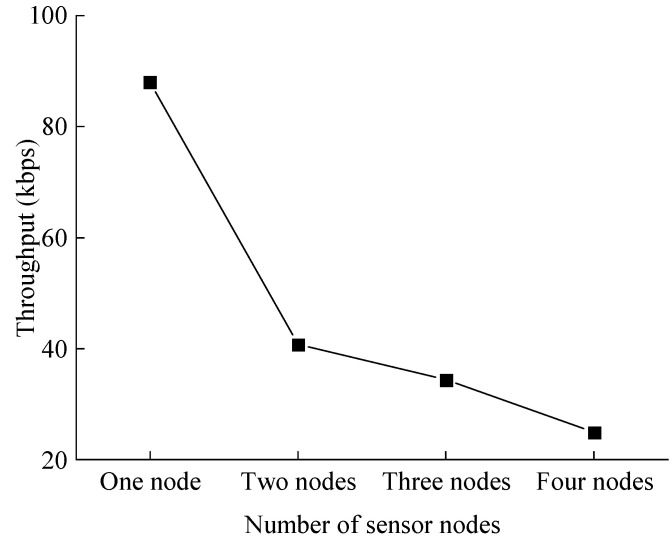
The throughput with increasing sensor node.

Theoretically, the bandwidth of the MvWSNs node is 250 kbps, i.e., 31.25 kB/s. However, due to the influence of the network protocol and data packet structure, the maximum throughput is only 163 kbps. In practice, the maximum achievable throughput is 100 kbps, which is 12.5 kB/s. In our experiments, 1024 points are set to calculate the monitoring result. For the MvWSNs node, a 24-bit ADC is occupied, so 3 Bytes of memory storage are needed for one point. Therefore, the total packet to be transmitted towards the monitoring center can be up to 3072 Bytes. In theory, the maximum data size is 127 Bytes, according to IEEE 802.15.4 [30]. The available payload is 100 Bytes in each data frame. In addition, the data information, including packet number and node number, is fixed in the front of the data frame and the final frame is 96 Bytes for each packet. Therefore, the total number of data packets that need to be transmitted is 3072/96 = 32. The time consumption for raw data transmission is 240 ms. Note that it is tested under the condition of only one MvWSNs node in the network. The transmission throughput of node 1 with an increasing number of MvWSNs nodes is depicted in Figure 11.

As Figure 11 shows, the transmission throughput is decreasing with an increasing number of sensor nodes. Further analysis of the transmission throughput reveals that throughput decreases by nearly half as nodes increase. This is because each node has equal access to the channel to transmit data. Although the time consumption for one sensor node to transmit 1024 points is double compared to the time consumption of the calculation process for the modified MobileNet, both of them are controlled within one second. The accumulated time consumption will increase. Furthermore, there are always at least four monitoring points. Therefore, the proposed method is time-saving and energy-saving.

## 5. Conclusions

Due to increased computational expenses and demanding resources from the numerous model parameters, DL is less affordable for embedded devices. However, the increase in data transmission urgently requires the integration of edge computing and DL for MvWSNs. In this article, we have proposed a lightweight rotating machinery fault detection approach through transplanting a MobileNet-based model to source-constrained edge devices. The well-trained model is implemented on the MvWSNs, a platform developed by STM32, to evaluated its performance. The performance of the proposed network on MvWSNs sensor nodes is with an acceptable accuracy of 0.98. Regarding the data transmission size, this method has the capacity to substantially reduce it to only 0.1% of the original data. The study introduces a new edge computing paradigm by combining the DL and IoT devices for applications such as high-speed trains and wind turbines.

Even though the proposed model shows good performance on sensor nodes, it cannot handle increasing sample rates. Furthermore, the comprehensive condition of the monitoring equipment is always evaluated using all the sampled data.

In the future, it would be interesting to develop a more efficient and lightweight model by considering the complex operations and the various sensor signals, such as temperature and pressure. to determine a comprehensive health condition for rotating machinery. Future studies should also consider how to integrate all the sampled data and the interpretability of the proposed model.

## Figures and Tables

**Table 1:** The network structure of fault diagnosis classification model based on MobileNet.

Layer	Input Shape
Convolution/2	(32, 32, 1)
Depth-wise convolution/1	(16, 16, 8)
Point-wise convolution/1	(16, 16, 16)
Depth-wise convolution/2	(8, 8, 16)
Point-wise convolution/2	(8, 8, 32)
Depth-wise convolution/1	(8, 8, 32)
Point-wise convolution/1	(8, 8, 64)
Depth-wise convolution/2	(4, 4, 64)
Point-wise convolution/2	(4, 4, 128)
Average pooling/1	(1, 1, 128)
Fully connected	(1, 1, 128)
Softmax	(1, 1, 5)

**Table 2:** The parameter of the MobileNet implemented on the MvWSN node.

Parameter	Size
Size of input data	1024 (4 kB)
Size of output data	5 (20 B)
Complexity	424,944 times
Size of weight	52,180 B
Size of activation function	20,994 B
Requirement of Flash memory	50.94 kB
Requirement of RAM memory	24.52 kB

**Table 3:** Description of the DDS.

Gearbox Category	Level	Gear Category	Number of Teeth	Number of Gears	Gear Ratio
Planetary gearbox	1st	Sun gear	20	1	6
		Inner gear ring	100	1	
		Planetary gear	40	3	
	2nd	Sun gear	28	1	4.57
		Inner gear ring	100	1	
		Planetary gear	36	3	
Fixed shaft gearbox	1st	Driving gear	100	1	0.29
		Drive gear	29	1	
	2nd	Driving gear	36	1	2.5
		Drive gear	90	1	

**Table 4:** Description of the health states.

Class Label	Health States Description
0	Normal
1	Tooth surface pitting fault of gear
2	Tooth root crack fault of gear
3	Outer fault of bearing
4	Inner fault of bearing

**Table 5:** The test accuracy of five conditions.

Condition	Accuracy
N	99.9%
F1	99.3%
F2	99%
F3	98.4%
F4	98.9%

## Data Availability

Data are contained within the article.

## References

[1] Ganga D., Ramachandran V. (2018). IoT-Based Vibration Analytics of Electrical Machines. IEEE Internet Things J..

[2] Pathinarupothi R.K., Durga P., Rangan E.S. (2019). IoT-Based Smart Edge for Global Health: Remote Monitoring with Severity Detection and Alerts Transmission. IEEE Internet Things J..

[3] Xiang S., Qin Y., Luo J., Wu F., Gryllias K. (2023). A concise self-adapting deep learning network for machine remaining useful life prediction. Mech. Syst. Signal Process..

[4] Yan T., Lei Y., Wang B., Han T., Si X., Li N. (2020). Joint maintenance and spare parts inventory optimization for multi-unit systems considering imperfect maintenance actions. Reliab. Eng. Syst. Saf..

[5] Zhu Z., Lei Y., Qi G., Chai Y., Mazur N., An Y., Huang X. (2023). A review of the application of deep learning in intelligent fault diagnosis of rotating machinery. Measurement.

[6] Du W., Guo Z., Li C., Gong X., Pu Z. (2022). From Anomaly Detection to Novel Fault Discrimination for Wind Turbine Gearboxes with a Sparse Isolation Encoding Forest. IEEE Trans. Instrum. Meas..

[7] Lee S.B., Stone G.C., Antonino-Daviu J., Gyftakis K.N., Strangas E.G., Maussion P., Platero C.A. (2020). Condition Monitoring of Industrial Electric Machines: State of the Art and Future Challenges. IEEE Ind. Electron. Mag..

[8] Liu P., Zhang Y., Wu H., Fu T. (2020). Optimization of Edge-PLC-Based Fault Diagnosis with Random Forest in Industrial Internet of Things. IEEE Internet Things J..

[9] Wang T.-Y., Meng J.-L., Li Q.-X., He Z.-Y., Zhu H., Ji L., Sun Q.-Q., Chen L., Zhang D.W. (2021). Reconfigurable optoelectronic memristor for in-sensor computing applications. Nano Energy.

[10] Fordal J.M., Schjølberg P., Helgetun H., Skjermo T.Ø., Wang Y., Wang C. (2023). Application of sensor data based predictive maintenance and artificial neural networks to enable Industry 4.0. Adv. Manuf..

[11] Rubes O., Chalupa J., Ksica F., Hadas Z. (2021). Development and experimental validation of self-powered wireless vibration sensor node using vibration energy harvester. Mech. Syst. Signal Process..

[12] He C., Han P., Lu J., Wang X., Song J., Li Z., Lu S. (2023). Real-Time Fault Diagnosis of Motor Bearing via Improved Cyclostationary Analysis Implemented onto Edge Computing System. IEEE Trans. Instrum. Meas..

[13] Tarokh M.H., El Houssaini D., Viehweger C., Kanoun O. (2021). Design of a Wireless Sensor Node Based on MSP430FR5969 for Environment Monitoring Applications, Proceedings of the 2021 18th International Multi-Conference on Systems, Signals & Devices (SSD), Monastir, Tunisia, 22–25 March 2021.

[14] Wang J., Gao Y., Liu W., Sangaiah A.K., Kim H.-J. (2019). An intelligent data gathering schema with data fusion supported for mobile sink in wireless sensor networks. Int. J. Distrib. Sens. Netw..

[15] Huang Y., Zhao C., Tang B., Yang Y., Fu H. (2022). Sparse Random Reconstruction of Data Loss with Low Redundancy in Wireless Sensor Networks for Mechanical Vibration Monitoring. IEEE Sens. J..

[16] Wu J., Tang T., Chen M., Wang Y., Wang K. (2020). A study on adaptation lightweight architecture based deep learning models for bearing fault diagnosis under varying working conditions. Expert Syst. Appl..

[17] Mekonnen Y., Namuduri S., Burton L., Sarwat A., Bhansali S. (2020). Review—Machine Learning Techniques in Wireless Sensor Network Based Precision Agriculture. J. Electrochem. Soc..

[18] Sodhro A.H., Pirbhulal S., de Albuquerque V.H.C. (2019). Artificial Intelligence-Driven Mechanism for Edge Computing-Based Industrial Applications. IEEE Trans. Ind. Inform..

[19] Park D., Kim S., An Y., Jung J.-Y. (2018). LiReD: A light-weight real-time fault detection system for edge computing using LSTM recurrent neural networks. Sensors.

[20] Lu S., Qian G., He Q., Liu F., Liu Y., Wang Q. (2020). In Situ Motor Fault Diagnosis Using Enhanced Convolutional Neural Network in an Embedded System. IEEE Sens. J..

[21] Malviya V., Mukherjee I., Tallur S. (2022). Edge-Compatible Convolutional Autoencoder Implemented on FPGA for Anomaly Detection in Vibration Condition-Based Monitoring. IEEE Sens. Lett..

[22] Huang Y., Tang B., Deng L., Zhao C. (2020). Fuzzy Analytic Hierarchy Process-Based Balanced Topology Control of Wireless Sensor Networks for Machine Vibration Monitoring. IEEE Sens. J..

[23] Sinha D., El-Sharkawy M. Thin mobilenet: An enhanced mobilenet architecture. Proceedings of the 2019 IEEE 10th Annual Ubiquitous Computing, Electronics & Mobile Communication Conference (UEMCON).

[24] Huang Q., Ding H., Effatparvar M. (2024). Breast cancer diagnosis based on hybrid SqueezeNet and improved chef-based optimizer. Expert Syst. Appl..

[25] Yang H., Liu J., Mei G., Yang D., Deng X., Duan C. (2023). Research on real-time detection method of rail corrugation based on improved ShuffleNet V2. Eng. Appl. Artif. Intell..

[26] Yu W., Lv P. (2021). An end-to-end intelligent fault diagnosis application for rolling bearing based on MobileNet. IEEE Access.

[27] Pham M.T., Kim J.-M., Kim C.H. (2020). Deep learning-based bearing fault diagnosis method for embedded systems. Sensors.

[28] Yao D., Li G., Liu H., Yang J. (2021). An intelligent method of roller bearing fault diagnosis and fault characteristic frequency visualization based on improved MobileNet V3. Meas. Sci. Technol..

[29] Crocioni G., Pau D., Delorme J.-M., Gruosso G. (2020). Li-ion batteries parameter estimation with tiny neural networks embedded on intelligent IoT microcontrollers. IEEE Access.

[30] Jardosh S., Ranjan P., Rawal D. Prioritized IEEE 802.15.4 for wireless sensor networks. Proceedings of the IEEE Wireless Advanced.

